# Relation between global end-diastolic volume and left ventricular end-diastolic volume

**DOI:** 10.1186/cc14255

**Published:** 2015-03-16

**Authors:** A Pironet, P Morimont, S Kamoi, N Janssen, PC Dauby, JG Chase, B Lambermont, T Desaive

**Affiliations:** 1 University of Liège, Belgium; 2 University of Canterbury, Christchurch, New Zealand

## Introduction

Measurement of global end-diastolic volume (GEDV) is provided by cardiovascular monitoring devices using thermodilution procedures. The aim of this study was to assess the relation between this clinically available index and left ventricular end-diastolic volume (LVEDV), which is typically not available at the patient bedside.

## Methods

Measurements were performed on six anaesthetized and mechanically ventilated pigs. Volume loading via successive infusions of saline solution was first performed and was followed by dobutamine infusion. These two procedures provided a wide range of LVEDV values. During these experiments, GEDV was intermittently measured using the PiCCO monitor (Pulsion AG, Germany) during thermodilutions and LVEDV was continuously measured using an admittance catheter (Transonic, NY, USA) inserted in the left ventricle.

## Results

Table 1 presents the linear correlations obtained between LVEDV and GEDV. These correlations are good to excellent, with *r*^2^ values from 0.59 to 0.85. However, the coefficients of the linear regressions present a large intersubject variability, which prevents the precise estimation of LVEDV using GEDV. Nevertheless, variations in LVEDV are well reproduced by the GEDV index. The variations in LVEDV actually equal 21 to 48% of those in GEDV. The coefficient *b *is always nonzero, indicating that some proportion of the GEDV index is actually not linked to LVEDV.

**Table 1:** Linear regressions between LVEDV and GEDV.

Subject	*a *	*b *(ml)	*r*2
1	0.26	7.64	0.82
2	0.43	-47.10	0.66
3	0.21	-12.99	0.75
4	0.25	-11.42	0.59
5	0.41	-65.42	0.85
6	0.48	-65.75	0.68

LVEDV = *a *× GEDV + *b*.

## Conclusion

The results show that GEDV and LVEDV are generally well correlated, but the correlation coefficients are subject specific. A preliminary calibration step (for instance using echocardiography) is thus necessary to infer LVEDV from GEDV.

